# Supplementary material for: Association between oral microbiome and seven types of cancers in East Asian population: a two-sample Mendelian randomization analysis
Source: Front Mol Biosci. 2023 Nov 21;10:1327893. doi: 10.3389/fmolb.2023.1327893 (PMC10702768; doi:10.3389/fmolb.2023.1327893)
Supplement: Supplementary file 1 [file DataSheet1.ZIP › Supplementary Materials/MR plots of saliva/saliva═╝/Breast cancer/pheno.125_to_breast cancer_leave_one_out.pdf]

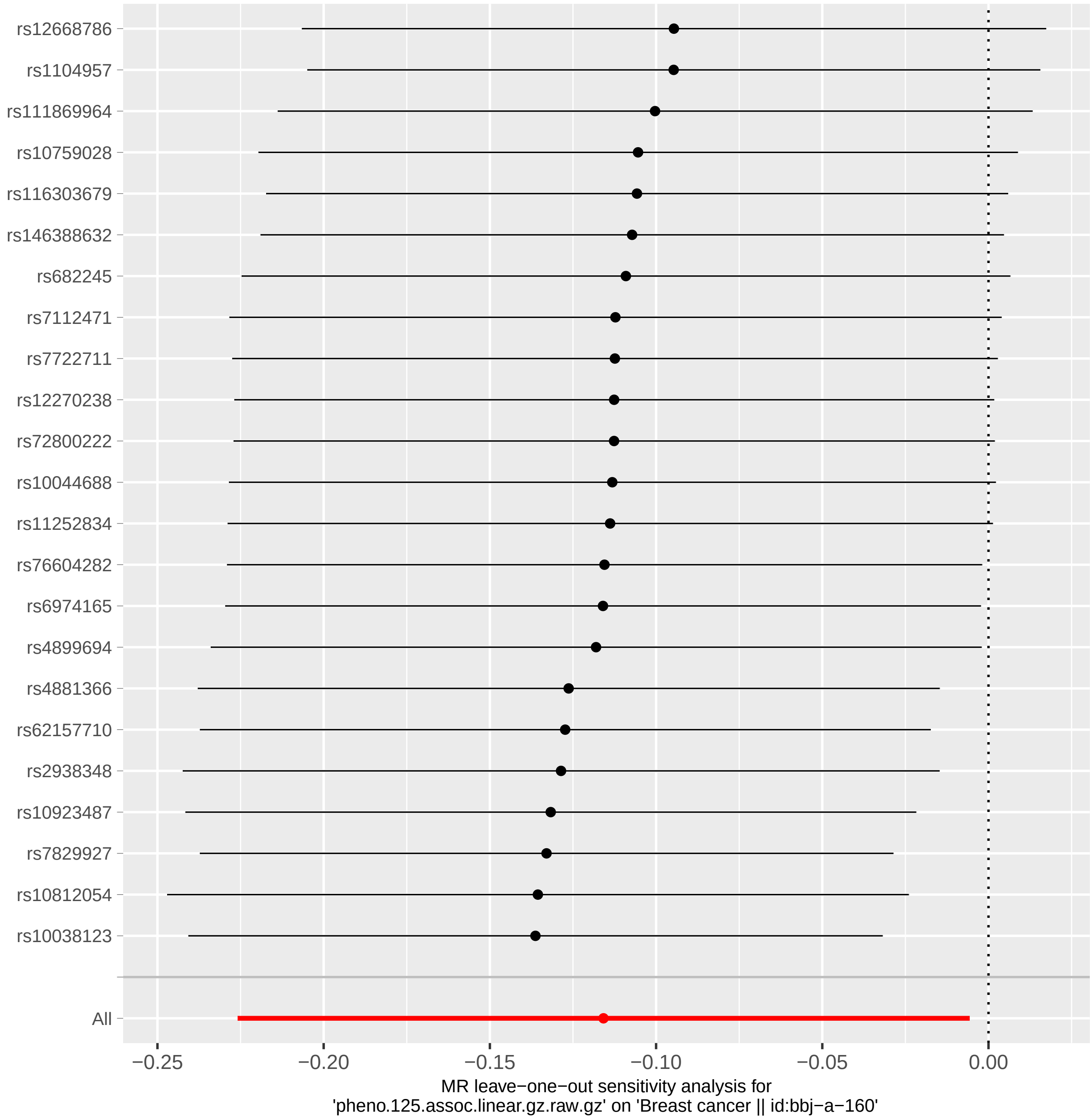

rs12668786
rs1104957
rs111869964
rs10759028
rs116303679
rs146388632
rs682245
rs7112471
rs7722711
rs12270238
rs72800222
rs10044688
rs11252834
rs76604282
rs6974165
rs4899694
rs4881366
rs62157710
rs2938348
rs10923487
rs7829927
rs10812054
rs10038123
All
−0.25
−0.20
−0.15
−0.10
−0.05
0.00
MR leave−one−out sensitivity analysis for
'pheno.125.assoc.linear.gz.raw.gz' on 'Breast cancer || id:bbj−a−160'
